# Genetic control of reproduction in dairy cows under grazing conditions

**DOI:** 10.21451/1984-3143-AR2018-0054

**Published:** 2018-08-03

**Authors:** Stephen T. Butler, Stephen G. Moore

**Affiliations:** Teagasc, Moorepark AGRIC, Fermoy, Co. Cork, Ireland

**Keywords:** fertility phenotypes, genetic merit, pasture.

## Abstract

Fertility performance is a key driver of the efficiency and profitability of seasonal-calving pasture- based systems of milk production. Since the 1990’s and early 2000’s, most countries have placed varying levels of emphasis on fertility and survivability traits, and phenotypic performance has started to improve. In recent years, the underlying physiological mechanisms responsible for good or poor phenotypic fertility have started to be unravelled. It is apparent that poor genetic merit for fertility traits is associated with multiple defects across a range of organs and tissues that are antagonistic to achieving satisfactory fertility performance. The principal defects include excessive mobilisation of body condition score (BCS), unfavourable metabolic status, delayed resumption of cyclicity, increased incidence of endometritis, dysfunctional estrous expression, and inadequate luteal phase progesterone concentrations. At a tissue level, coordinated changes in gene expression in different tissues have been observed to orchestrate more favourable BCS, uterine environment and corpus luteum function. Interestingly, cows with poor genetic merit for fertility traits have up-regulated inflammation and immune response pathways in multiple tissues. Sire genetic merit for daughter fertility traits is improving rapidly in the dairy breeds, especially in the predominant Holstein and Friesian breeds. With advances in animal breeding, especially genomic technologies to identify superior sires, genetic merit for fertility traits can be improved much more quickly than they initially declined.

## Introduction

*“The finest trick of the devil is to persuade you that he does not exist”*

Charles Baudelaire (9 April 1821 - 31 August 1867).

Genetic gain in dairy cow milk production during the last century has been impressive, highlighting the success that can be achieved through intensive selection on traits of interest. In large part, the genetic gain was achieved by selecting exclusively on milk production traits, and ignoring other functional traits (health, fertility). The foregoing thinking was that selection for daily milk production would be more successful if intensively selected (true), and that improvements in management could adequately compensate for any deterioration in genetic merit for fertility traits (in hindsight, not true). Eventually, this led to a marked decline in both genetic merit for fertility traits and phenotypic fertility performance (Pryce *et al*., 2014). The belief that a genetic influence on phenotypic fertility does not exist is clearly no longer valid. We now know that genetic background has a strong influence on phenotypic fertility performance, but as a complex trait, there are many genes that have an influence, each with very small effect (Berry *et al*., 2014; Pryce *et al*., 2014).

A compact calving pattern, with most animals calving within six weeks after the planned start of calving, is a cornerstone of efficient seasonal pasture- based milk production (Butler, 2014). Achieving this compact calving pattern necessitates excellent fertility performance during a compact breeding period. The deterioration in phenotypic fertility reached its zenith in the late 1990’s and early 2000’s in many countries that operate seasonal-calving pasture-based systems, resulting in longer breeding periods, spread out calving patterns and greater culling due to fertility failure (Evans *et al*., 2006). During the last twenty years, there has been renewed focus on selecting for improved fertility traits, with recent trends indicating marked improvements in both genetic merit for fertility traits and phenotypic fertility performance in many countries (Pryce *et al*., 2014). Between 1990 and 2000, average calving interval increased by 1.25 days per year, but since the mid-2000s calving intervals have plateaued or decreased in many countries (Pryce *et al*., 2014). Major gene effects on cow fertility have been previously reviewed (Butler, 2013). This review will describe the cow fertility phenotypes that are under genetic control in pasture-based lactating dairy cows.

## Selecting for Improved Fertility

The initial selection indices in most dairy countries focused primarily on milk production traits (Cole and VanRaden, 2018). In addition to selecting for cows that produced more milk, there was also a focus on ‘dairy type’, meaning that greater angularity or sharpness was also considered favourable (i.e., cows also *looked* like they produced more milk). Many studies in different systems of production have indicated that body condition score (BCS) is a key driver of cow health and fertility (Berry *et al*., 2003; Buckley *et al*., 2003; Lucy, 2003; Weigel, 2006; Roche *et al*., 2009; Cummins *et al*., 2012c; Fenlon *et al*., 2017). Favourable BCS, however, is the opposite of favourable angularity. It is likely that selecting for angularity directly contributed to the decline in phenotypic fertility and increased the incidence of metabolic disorders (Hansen, 2000). Selecting for improved BCS has been identified as a strategy to improve health and fertility (Berry *et al*., 2003; Weigel, 2006). The advent of automated technologies to facilitate frequent low-cost collection of BCS records from large numbers of cows could facilitate incorporation of this phenotype into selection indexes.

For many decades, fertility and health traits (most notably mastitis) have been incorporated into the breeding index of the Scandinavian breeds. While fertility globally declined between 1985 and 2005, non- return rates, culling rates due to infertility and calving interval remained relatively constant in the Norwegian Red breed (Refsdal, 2007). The incidence of veterinary treatments for reproductive disorders in 503,683 first- lactation daughters of 1,058 Norwegian Red sires was 3.1% for silent heats, 0.9% for metritis, 0.5% for cystic ovaries, and 1.5% for retained placenta (Heringstad, 2010). The low incidence of fertility disorders and maintenance of high phenotypic fertility performance provide support for the objective of selecting for improved fertility. In the US, a specific index for grazing herds was developed in 2014 (Gay *et al*., 2014), with relative index weights on productive life (7%) and daughter pregnancy rate (20%) that were a reversal of the weights on these traits in the net merit index at that time (19% and 7%, respectively) (VanRaden, 2017). The greater emphasis on daughter pregnancy rate reflects the increased importance of phenotypic fertility in seasonal-calving grazing systems than in year-round calving systems, whereas less emphasis on productive life is required in grazing systems because less production per cow generally results in longer survival.

In Ireland, liberalisation of semen importation regulations and intense selection for milk production traits lead to the introgression of North American Holstein genes into the predominantly British Friesian national herd. During the period from 1990 to 2001, genetic merit for milk yield increased by 25 kg per year, the proportion of Holstein genes increased from 8% to 63% and the calving rate to first service declined from 55% in 1990 to 44% in 2001 (Evans *et al*., 2006). High milk production North American Holstein cows were bred for a confinement based system, where energy dense Total Mixed Ration diets were the standard feeding practice. In a grass based system, the energetic demands associated with milk production could not be met solely by grass dry matter intake (DMI), rendering the cows susceptible to excessive tissue mobilisation, negative energy balance, poor BCS and reproductive failure (Buckley *et al*., 2000a; Horan *et al*., 2004).

To address the problem of declining fertility, the Irish national breeding programme introduced a multi-trait selection index called the Economic Breeding Index (EBI) in 2001 (Veerkamp *et al*., 2002). This index included production and non-production traits, thus identifying sires of superior genetic merit for delivering on-farm profit. Since its introduction, the EBI has evolved to include 6 sub-indexes, with the fertility sub- index accounting for 35% of the relative index weight (ICBF, 2018). The fertility sub-index is comprised of 2 traits; calving interval (23.5%) and survival (11.6%).

## North American and New Zealand lactating cow strain-comparison studies

Several strain-comparison studies in Ireland and New Zealand compared lactating cows with North American (NA) and New Zealand (NZ) ancestry, and the main outcomes were discussed in a recent review (Butler, 2013). In general, these studies highlighted lower milk volume but similar milk solids production, greater BCS and reduced BCS loss, similar DMI per unit of metabolic body weight, similar or earlier commencement of luteal activity, greater insulin responsiveness, greater circulating insulin-like growth factor 1 (IGF1), similar or greater hepatic IGF1 expression, greater endometrial expression of genes associated with (i) immune tolerance to the embryo, (ii) prevention of luteolysis, and (iii) embryo support and development, and superior reproductive performance for the NZ Holstein-Friesian compared with NA Holstein- Friesian (Harris and Kolver 2001; Horan *et al*., 2004, 2005; McCarthy *et al*., 2007; Patton *et al*., 2008; Lucy *et al*., 2009; McCarthy *et al*., 2009; Walker *et al*., 2012). Data from these studies collectively suggest that early lactation adaptations may have more adverse effects in the NA compared with the NZ strain of dairy cow, and that this is likely related to their greater genetic potential for milk volume.

## Fert+ and Fert- lactating cow genetic model of fertility

Cows with high genetic merit for milk production have generally been reported to have poorer fertility than cows with average genetic merit for milk production (Lucy and Crooker, 1999; Buckley *et al*., 2000; Horan *et al*., 2004). It is unlikely; however, that high phenotypic milk production *per se* is directly responsible for poor fertility. A number of studies have indicated similar or even superior fertility in high yielding cows compared to lower yielding cows (Nebel and McGilliard, 1993; Gröhn and Rajala-Schultz, 2000; Bello *et al*., 2013). As a result, it is difficult to identify specific mechanisms under genetic control responsible for poor fertility using animal models that differ in phenotypic milk production potential in addition to a wide range of associated phenotypes (milk composition, body weight, feed intake capacity, etc.).

To address this issue, a lactating cow model with similar genetic merit for milk production, but either good (Fert+) or poor (Fert-) genetic merit for fertility traits was recently developed and validated at Moorepark (Cummins *et al*., 2012a). These cows have similar proportions of Holstein genetics, and similar body weight, milk yield and milk composition. Fertility performance, however, is markedly poorer in the Fert- cows compared to the Fert+ cows (Cummins *et al*., 2012a). A series of experiments was performed to characterise uterine health during uterine involution, follicular and luteal growth, reproductive hormone concentrations, behavioural estrus during the estrous cycle, metabolic status and control of nutrient partitioning during lactation in Fert+ and Fert- cows.

The research conducted to date with this animal model has clearly demonstrated that the causes of reduced fertility in the Fert- cows are multifactorial.

## Uterine health

The reproductive tract of all cows becomes exposed to microbial pathogens while the cervix remains open after delivery of the fetal-placental unit. The development of uterine disease depends on the type of bacteria involved and on the immune response of the cow, and is associated with reduced subsequent fertility (Sheldon *et al*., 2009). We recorded vaginal discharge scores weekly after calving to assess the temporal changes in clinical endometritis, and also examined uterine cytology at three and six weeks postpartum to assess subclinical endometritis (Moore *et al*., 2014a). The vaginal discharge scores and uterine cytology results indicated greater incidence of clinical and subclinical endometritis in the Fert- cows, respectively. These findings indicate that the Fert+ cows were capable of mounting a stronger and/or timelier immune response following exposure to microbial pathogens. Endometritis adversely affects the local uterine environment, but also indirectly affects fertility through altered follicle development and function (Sheldon *et al*., 2002), and post-ovulatory effects on corpus luteum development (Williams *et al*., 2007).

## The estrous cycle

The estrous cycle of lactating cows was synchronized when cows were approximately 80 to 100 days postpartum. The estrous synchronization protocol lasted 10 days [day 0: i.m. GnRH and insertion of P4 device; day 7: i.m. PGF2α; day 8 removal of P4 insert; day 10: expected day of estrus]. Ultrasound exams and blood sample collection were conducted daily beginning on the expected day of estrus (See Cummins *et al*. (2012b) for details). The estrous cycle was 4.1 days longer in Fert- cows compared with Fert+ cows (25.1 vs. 21.0 days; P = 0.01), and this was associated with Fert- cows tending to have more follicular waves (2.7 vs. 2.2; P = 0.07). Circulating progesterone (P4) concentrations were similar during the first five days of the estrous cycle, but from day 5 to day 13, circulating P4 concentrations were 34% greater in Fert+ cows (5.15 vs. 3.84 ng/mL; P < 0.001). The difference in circulating P4 was associated with a 16% larger CL volume in Fert+ cows. A follow-up study also detected greater circulating P4 concentrations in Fert+ cows, but failed to detect differences in metabolic clearance rate of P4 or hepatic mRNA abundance of genes responsible for P4 catabolism (*CYP2C, CYP3A, AKR1C* family; Moore *et al*. (2014b). This suggests that the greater circulating P4 concentrations in Fert+ cows is primarily a result of greater luteal P4 synthetic capacity (larger CL size and greater P4 output per unit of CL tissue). The effects of circulating P4 may be manifest pre- and post-ovulation. A large volume of literature supports the pivotal role of P4 on the preovulatory oocyte and follicle (Inskeep, 2004), from day 5 to 13 of the estrous cycle to influence functional changes in histotroph composition (Green *et al*., 2005), structural changes in endometrial glandular duct density (Wang *et al*., 2007), endometrial gene expression (Forde *et al*., 2009), maternal recognition of pregnancy (Mann and Lamming, 2001) and likelihood of subsequent pregnancy establishment (Herlihy *et al*., 2013). Inherent differences in circulating P4 concentrations likely represent a key phenotype responsible for fertility differences between these two strains.

## Estrous behaviour

Estrous behaviour (measured using automated activity meters and electronic mount detectors) and the timing of ovulation (measured using transrectal ultrasound) were recorded at a synchronised estrus and the subsequent spontaneous estrus (Cummins *et al*., 2012b). Fert- cows had a greater incidence of silent heats (i.e., ovulation in the absence of behavioural estrus) at the end of the synchronised estrous cycle. A greater proportion of Fert- cows also displayed behavioural signs of estrus, but subsequently failed to ovulate. Of the estrus events recorded, 36% fell into the combined categories of silent heats and heats without ovulation in Fert- cows, whereas only 2% fell into the combined categories in Fert+ cows. As the Fert+ cows have been repeatedly observed to have greater luteal phase P4 concentrations (Cummins *et al*., 2012b; Moran *et al*., 2015; Moore *et al*., 2016), differences in luteal- phase P4 priming of the neural mechanisms involved in estrous behaviour and GnRH release could explain some of the differences in estrus behaviour between Fert+ and Fert- cows. It is possible that sub-optimal P4 concentrations in the estrous cycle pre-breeding interferes with the normal endocrine feedback mechanisms that are required to facilitate appropriately timed estrous behaviour and ovulation.

## Endometrium - corpus luteum interaction

The differences in circulating P4 concentrations between Fert+ and Fert- cows during the luteal phase prompted an investigation into the simultaneous gene expression profile in the corpus and the endometrium (Moore *et al*., 2016). Cows were synchronised, blood samples were collected daily, periodic ultrasound exams were conducted to assess the corpus luteum development, and biopsies of the corpus luteum and endometrium were collected on day 13 post- estrus. Once again, CL volume and circulating progesterone concentrations were greater in Fert+ cows compared with Fert- cows. Global transcriptomics of the endometrium indicated greater inflammation, less favourable cellular energy status and greater synthesis and secretion of prostaglandin F2α in Fert- cows. Global transcriptomics of the corpus luteum indicated greater PGF2α response, and lesser steroidogenesis, and mRNA processing in Fert- cows. Hence, coordinated communication between the corpus luteum and the endometrium was evident, highlighting the exquisite regulation necessary to facilitate pregnancy establishment.

## Metabolic status and BCS

Fert+ cows maintain greater postpartum BCS, which is facilitated by greater DMI (Moore *et al*., 2014a). Differences in metabolites and metabolic hormones are broadly reflective of better metabolic status. Circulating concentrations of IGF1 are greater in Fert+ cows throughout lactation (Cummins *et al*., 2012a). Despite Fert+ cows having greater circulating IGF1 concentrations, hepatic *IGF1* gene expression is greater only in mid to late-lactation (Cummins *et al*., 2012c). The half-life of IGF1 in circulation is ~10 minutes as a free peptide, ~30 to 90 minutes when bound to a low molecular weight binding protein (IGFBP2, IGFBP4, IGFBP5 and IGFBP6), and 12 to 15 hours in the ternary complex of IGF1, IGFBP3 and insulin-like growth factor binding protein, acid labile subunit (Jones and Clemmons, 1995). Fert+ cows had reduced expression of low molecular weight binding proteins during early lactation (Cummins *et al*., 2012c), allowing longer IGF1 half-life in the ternary complex. Fert+ cows have greater circulating concentrations of insulin and glucose during the immediate postpartum period (Cummins *et al*., 2012a; Moore *et al*., 2014a). Elevated circulating concentrations of glucose in the peripartum period increased the likelihood of early ovulation (Butler *et al*., 2006) and conception at breeding (Garverick *et al*., 2013).

## Cellular Control of Nutrient partitioning

Hepatic and muscle transcriptomics were examined in Fert+ and Fert- cows during late pregnancy (LP), early lactation (EL) and mid-lactation (ML) to examine the molecular mechanisms that underpin the observed differences in BCS (Moran *et al*., 2016). We found 807 and 815 unique genes to be differentially expressed in at least one time-point in liver and muscle respectively, of which 79% and 83% were only found in a single time-point; 40 and 41 genes were found differentially expressed at every time-point, possibly indicating chronic dysregulation. We found 402, 338 and 282 genes differentially expressed in liver and 262, 527 and 212 genes differentially expressed in muscle at LP, EL and ML, respectively. Across all three time points, the differentially expressed genes pointed to the biological theme ‘*metabolism, lipid and carbohydrate’*, and specific functional annotation groups that were detected included ‘*gluconeogenesis*’ and ‘*extra-cellular growth factor*’ during late pregnancy, ‘*biosynthetic process*’, ‘*lipid lipoprotein*’ and ‘*metabolic process*’ during early lactation, and ‘*lipid*’ and ‘*lipoprotein particle*’ during mid-lactation. The collective findings indicated key differences at each stage of lactation: (1) Fert+ cows were less reliant on mobilised muscle tissue as a source of glucose precursors and mobilised fat for cellular energy requirements during LP; (2) in EL, Fert+ cows had greater hepatic gluconeogenic capacity; and (3) in ML Fert+ cows had greater hepatic *IGF1* expression as well as up-regulation of fatty acid synthesis pathways. Clearly, the ability of Fert+ cows to maintain superior BCS and similar milk energy output compared with the Fert- cows is dependent on orchestrated changes involving multiple tissues, including liver and muscle, indicating better homeorhetic adaptation to lactation.

## Inflammation

A notable observation from multiple studies that examined global transcriptomics in biopsy samples of liver, muscle, endometrium and corpus luteum collected from Fert+ and Fert- cows was differences in immune and inflammation pathways. In liver and muscle biopsies, differentially expressed genes at LP, EL and ML time points pointed toward the biological theme *‘immune and inflammatory’* processes, and were generally up-regulated in Fert- cows. Specific annotation terms identified included ‘chemokine’ and ‘MHC complex’ in LP, ‘defense response’ and ‘immunoglobuiln’ in EL, and ‘acute phase response’ in ML (Moran *et al*., 2016). In endometrium tissue samples collected on day 7 (Moran *et al*., 2015) or on day 13 (Moore *et al*., 2016) post-estrus, the acute-phase protein serum amyloid A3 (*SAA3*) was up-regulated in Fert- cows, which has been reported to be highly induced in bovine endometrium in response to Escherichia coli infection (Chapwanya *et al*., 2013). This finding was consistent with greater incidence of clinical and sub-clinical endometritis in Fert- cows (Moore *et al*., 2014a). *SAA3* expression was also up- regulated in Fert- cows in corpus luteum tissue samples collected on day 13 post-estrus (Moore *et al*., 2016).

## Conclusions

The main phenotypes that are different between cows with good and poor genetic merit for fertility traits are summarized in Table 1. Their contribution and relative importance to overall reproductive importance is likely collaborative rather than independent. Numerous genome-wide association studies consistently illustrate the multifactorial nature of bovine fertility (Berry *et al*., 2014). The impact of genetic selection programmes on improved dairy cow fertility over the past decade is supported by an abundance of scientific literature demonstrating only minor and inconsistent effects from nutritional supplementation (Roche *et al*., 2011; Butler, 2014) and hormonal manipulation (Bisinotto *et al*., 2015). Importantly, well-established phenotypes (BCS, estrous behaviour, hormone concentrations) associated with dairy cow fertility (Walsh *et al*., 2011) are under genetic control and may become useful in fertility genetic evaluations if sufficient records become available. The prospect of automated monitoring of animal health, body condition score, ovarian activity, estrous behaviour, and milk hormone concentrations is quickly becoming a reality due to developments in milking automation, camera technology, activity monitors and in-line milk analysis. Access to large datasets of fertility phenotypes collected from diverse cow populations with genotype information may further enhance our ability to accurately identify QTL’s associated with reproductive efficiency and increase the rate of genetic gain. This approach was recently utilised to elucidate the genetic control of stature in cattle (Bouwman *et al*., 2018), and could also be successful for female fertility traits despite the low heritability. Considering the differences in reproductive management between confinement (reliance on hormonal treatment) and pasture-based (AI after spontaneous estrus) and the greater selection pressure placed on fertility in pasture-based systems, further investigation is warranted to determine if the genetic and physiological differences between fertility genotypes are conserved across production environments. Nevertheless, after many decades of declining fertility, genetic merit for fertility and phenotypic reproductive performance now appears to be on the opposite trajectory.

**Table 1:** Summary of the principal physiological mechanisms responsible for greater fertility in Fert+ cows compared with Fert- cows.

Early postpartum	Pregnancy establishment
Greater DMI	Stronger expression of estrus
Shorter postpartum anestrous interval	Fewer silent heats, and less incidence of ovulation failure after expression of estrus
Reduced incidence of clinical and subclinical endometritis	Greater luteal phase circulating P4
More favourable systemic indicators of metabolic status	Better coordination of corpus luteum and endometrium gene expression to support luteal P4 synthesis and endometrial receptivity
Better coordination of hepatic and peripheral tissue gene expression in support lactation and BCS maintenance	Better coordination of hepatic and peripheral tissue gene expression in support lactation and BCS maintenance
Less inflammation in liver and muscle	Less inflammation in liver, muscle, endometrium and corpus luteum
